# Reduced Serum Butyrylcholinesterase Activity Indicates Severe Systemic Inflammation in Critically Ill Patients

**DOI:** 10.1155/2015/274607

**Published:** 2015-02-11

**Authors:** Aleksandar R. Zivkovic, Karsten Schmidt, Annette Sigl, Sebastian O. Decker, Thorsten Brenner, Stefan Hofer

**Affiliations:** Department of Anesthesiology, Heidelberg University Hospital, Im Neuenheimer Feld 110, 69120 Heidelberg, Germany

## Abstract

Systemic inflammation is an immune response to a nonspecific insult of either infectious or noninfectious origin and remains a challenge in the intensive care units with high mortality rate. Cholinergic neurotransmission plays an important role in the regulation of the immune response during inflammation. We hypothesized that the activity of butyrylcholinesterase (BChE) might serve as a marker to identify and prognose systemic inflammation. By using a point-of-care-testing (POCT) approach we measured BChE activity in patients with severe systemic inflammation and healthy volunteers. We observed a decreased BChE activity in patients with systemic inflammation, as compared to that of healthy individuals. Furthermore, BChE activity showed an inverse correlation with the severity of the disease. Although hepatic function has previously been found essential for BChE production, we show here that the reduced BChE activity associated with systemic inflammation occurs independently of and is thus not caused by any deficit in liver function in these patients. A POCT approach, used to assess butyrylcholinesterase activity, might further improve the therapy of the critically ill patients by minimizing time delays between the clinical assessment and treatment of the inflammatory process. Hence, assessing butyrylcholinesterase activity might help in early detection of inflammation.

## 1. Introduction

Systemic inflammation is the immune response to a nonspecific insult of either infectious or noninfectious origin. The pathogenesis of systemic inflammation, closely related to sepsis, is not fully understood and remains a challenge in the intensive care unit (ICU) with high mortality rates (25–38%) [1, 2]. Systemic inflammation is a complex and dynamic process often associated with deleterious consequences [3]. More than 170 inflammation biomarkers have been described in the literature and proposed for the prognosis or diagnosis of the systemic inflammation or sepsis [4]. However, only a combined interpretation of the laboratory results and the clinical findings allows for an adequate and early therapy.

Cholinergic neurotransmission has been shown to play an important role in the regulation of the immune response during inflammation [5]. Increased Vagus nerve activity during inflammation inhibits peripheral cytokine release through a mechanism that requires nicotinic acetylcholine receptors [6]. The information from peripheral inflammatory responses is gathered through afferent fibers of the vagus nerve, followed by an instant efferent feedback, in a homeostatic fashion [7]. This mechanism has been described as “cholinergic anti-inflammatory pathway.” Indeed, our previous studies suggested that activation of cholinergic anti-inflammatory pathways by treatment with the cholinesterase inhibitor physostigmine during experimental endotoxemia might prove beneficial for systemic inflammation therapy [8, 9].

Cholinesterases are enzymes which hydrolyze the neurotransmitter acetylcholine. Butyrylcholinesterase (BChE), also known as “pseudo”-cholinesterase or “serum”-cholinesterase, is a nonspecific choline esterase, able to hydrolyze acetylcholine, as well as other esters. BChE is abundant in blood serum, pancreas, liver, and the central nervous system [10]. BChE is synthesized in the liver and has therefore been conventionally used as a liver function biomarker [11, 12]. Indeed, the work of al-Kassab and Vijayakumar [13] suggested the importance of BChE as an indicator of hepatic dysfunction in the septic syndrome. In addition, the role of BChE in lipid metabolism, obesity, and diabetes mellitus has been described [14, 15]; however, the exact physiological function of BChE remains unknown.

The study of Das [16] has suggested that an increase in BChE activity results in reduced serum and tissue acetylcholine levels, leading to disrupted cholinergic anti-inflammatory responses. Reduced cholinergic reaction would, in turn, amplify systemic inflammation creating a vicious circle.

Use of point-of-care-testing (POCT) in patient treatment has proven beneficial for the patient security and therapy. When compared to the conventional diagnostic laboratory analysis, the time interval between the analysis and results of a POCT approach is remarkably shorter, allowing for immediate decisions on further therapeutic and diagnostic procedures [17]. Indeed, early goal-directed therapy has been proven essential in the treatment of critically ill patients diagnosed with severe sepsis [18].

Here we show that serum BChE activity, measured by means of POCT, might be beneficial in early identification of severe systemic inflammation.

## 2. Materials and Methods

The observational clinical study was approved by the local ethics committee and was conducted in the surgical intensive care unit of the University Hospital of Heidelberg, Germany. All study patients or their legal designees signed written informed consent (Ethics Committee of the Medical Faculty of Heidelberg Trial-Code No. S-097/2013 and No. S-196/2014). In total, 80 individuals in two groups were enrolled in the study. The two groups included 40 patients diagnosed with severe systemic inflammation, according to the criteria of the Surviving Sepsis Campaign: International Guidelines for Management of Severe Sepsis and Septic Shock [19] and 40 healthy volunteers (the volunteer group) (Table 1). Identifying the precise starting time point of the systemic inflammation in critically ill patients is a complex procedure. Therefore, the inclusion criteria for the patient group, based on International Guidelines for Management of Severe Sepsis and Septic Shock, allowed for standardised and early diagnosis and therapy in the ICU environment. Patients were further classified into survivor and nonsurvivor subgroups, based on the 28-day survival analysis. The observation period of 28 days has been chosen according to the recommendations reported by [20]. It has been shown that primary diagnosis and the initial severity of illness during ICU stay strongly determine the acute prognosis in the early phase. The disease severity during ICU stay is, however, of almost no importance to long-term prognosis, which is mainly determined by preexisting conditions [21]. The management of septic patients in the intensive care unit included early goal-directed therapy [18], elimination of the septic focus and broad-spectrum antibiotics [22, 23]. As a control group, we chose 40 healthy volunteers without any signs of infection. Blood samples for the BChE analysis from patients diagnosed with systemic inflammation were collected once daily for up to 6 days. Relevant baseline data (demographic data, primary site of infection, and outcome) were collected. Severity of illness was estimated using the Simplified Acute Physiology Score (SAPS II), the Sequential Organ Failure Assessment score (SOFA), and Acute Physiology And Chronic Health Evaluation score (APACHE II). Patients with systemic inflammation were reevaluated for survival 28 days after enrolment in the study. Blood samples from the volunteer group were collected once.

After routine blood collection, as a part of the standardized ICU diagnostic and therapeutic procedure, 10 *μ*L of blood was taken for the BChE activity analysis. We used ChE Check (Securetec Detektions-Systeme AG, Neubiberg, Germany; In-Vitro-Diagnostics Guideline 98/79/EG; DIN EN ISO 18113-2 and -3), a point-of-care-testing device to determine the BChE activity according to the manufacturers' instructions. This is an enzymatic assay providing rapid and precise determination of BChE activity in human whole blood without any pretreatment of the samples. BChE activity is assayed by indirectly measuring the production of thiocholine from the hydrolysis of the specific substrate s-butyrylthiocholine iodide. Thiocholine reacts with 5,5′-dithio-bis-2-nitrobenzoic acid (DTNB, Ellman's reagent) as a chromogenic reagent, producing the yellow 5-thio-2-nitrobenzoate anion (TNB, Ellman's anion). The production of TNB was monitored at 470 nm [24]. Enzyme activity is expressed as U/L.

We used Cohen's *d*-test (*d* = 0.57; power 80%, significance level alpha = 5%) to determine the sample size. The resulting study data were entered into an electronic database (Microsoft Excel 2002, Microsoft Corp., Redmond, WA) and evaluated using GraphPad Prism version 6.0c for Mac (GraphPad Software, La Jolla California USA, http://www.graphpad.com/). Data were presented as median with interquartile range (IQR). Statistical significance was tested using a Mann-Whitney test. Correlation analysis was performed using Spearman's rank correlation test. *P* < 0.05 was considered statistically significant.

## 3. Results

Our study included 40 patients admitted to the surgical intensive care unit and diagnosed with systemic inflammation during their stay. Basic demographic and clinical data are listed in Table 1. Systemic inflammation was diagnosed by measuring C-reactive protein (CRP) levels, procalcitonin (PCT) levels, and white blood cell counts (WBCC) (Table 1). All patients showed significantly elevated inflammatory biomarkers, suggesting a severe systemic inflammation. Further diagnostics identified the primary site of the inflammatory focus in 34 (85%) of ICU patients, including 21 (53%) with gastrointestinal, 11 (27%) with respiratory, and 2 (5%) patients with genitourinary system focus (Table 1). Systemic inflammatory response syndrome (SIRS), an inflammation without identified infection, was diagnosed in 6 (15%) patients. To assess the disease severity we implemented APACHE II, SOFA, and SAPS II scores. The analysis showed midranged levels of the disease severity scores (Table 1). Outcome analysis revealed 31 patients (78%) as 28-day survivors.

**Figure 1:**
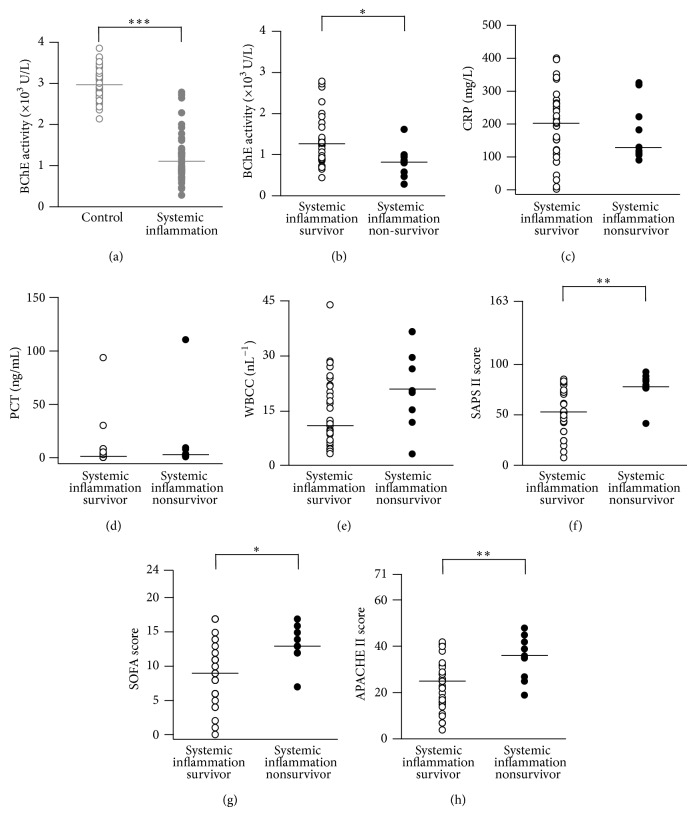
Reduced BChE activity in patients with systemic inflammation. (a) Scatter plots represent results of BChE point-of-care-testing (POCT) obtained from healthy volunteers (gray open circles, control) and patients diagnosed with systemic inflammation (gray closed circles, see Table 1). (b) Based on subsequent 28-day-survival analysis, patients were further divided into the survivor and nonsurvivor subgroups (black open and closed circles, resp.). ((c)–(h)) Histograms represent concurrent measurements of inflammation biomarkers CRP (*n* = 40 measurements (c)), PCT (*n* = 34 measurements (d)) and WBCC (*n* = 40 measurements (e)) and disease severity scores (SAPS II, SOFA, and APACHE II, *n* = 40 measurements each (f), (g), and (h)) from survivors (open circles) and nonsurvivors (closed circles). Bars are median values. ^*^ *P* < 0.05, ^**^ *P* < 0.01, and ^***^ *P* < 0.001 (Mann-Whitney test). BChE: butyrylcholinesterase; CRP: C-reactive protein; PCT: procalcitonin; WBCC: white blood cell count; SAPS: simplified acute physiology score; SOFA: sequential organ failure assessment; APACHE: acute physiology and chronic health evaluation.

By using a point-of-care-testing system we measured the activity of BChE from ICU patients with diagnosed systemic inflammation (see Table 1) and in 40 healthy volunteers. The results revealed lower BChE activity in patients with systemic inflammation, as compared to that of healthy individuals (1108 (585) U/L versus 2966 (526) U/L, *P* < 0.0001, Mann-Whitney test, Figure 1(a)).

Based on the 28-day-survival analysis, we further divided the patients into survival and nonsurvival subgroup. The within group analysis indicated an additional drop in the BChE activity observed in the nonsurvivor group as compared to survivors (820 (450) U/L versus 1268 (772) U/L, *P* < 0.05, Mann-Whitney test, Figure 1(b)). This finding suggests that BChE activity predicts mortality in patients with severe systemic inflammation. In addition, we compared the levels of inflammation biomarkers in the tested subgroups. Serum concentrations of CRP (reference interval < 5 mg/L) and PCT (reference interval < 0.05 ng/mL, measured every 2-3 days) [25] remained persistently elevated in survivor (CRP: 203 (154) mg/L; PCT: 1.6 (4.3) ng/mL) and nonsurvivor group (CRP: 130 (161) mg/L; PCT: 3.0 (7.3) ng/mL; Figures 1(c) and 1(d)). Surprisingly, slightly lower CRP levels were detected in the nonsurvivor group, as compared to the survivors. This might presumably be due to the fulminant course of the inflammatory disease, often observed in the nonsurvivor group. A trend towards an increased white blood cell count (WBCC) was observed in the nonsurvival group as compared to the survivor group (21 (19) nL^−1^ versus 11 (15) nL^−1^, *P* = 0.07, Mann-Whitney test, Figure 1(e)). To validate this finding, we assessed the disease severity by using disease classification systems: SAPS II, SOFA, and APACHE II scores. Indeed, the results showed significantly higher scores in the nonsurvivor as compared to the survivor group (SAPS II: 80 (11) versus 53 (31), *P* < 0.01; SOFA: 13 (4) versus 9 (6), *P* < 0.05; APACHE II: 36 (18) versus 25 (13), *P* < 0.01, Mann-Whitney test; Figures 1(f), 1(g), and 1(h)). The results suggest that reduction in BChE activity could be associated with fatal systemic inflammation.

**Figure 2:**
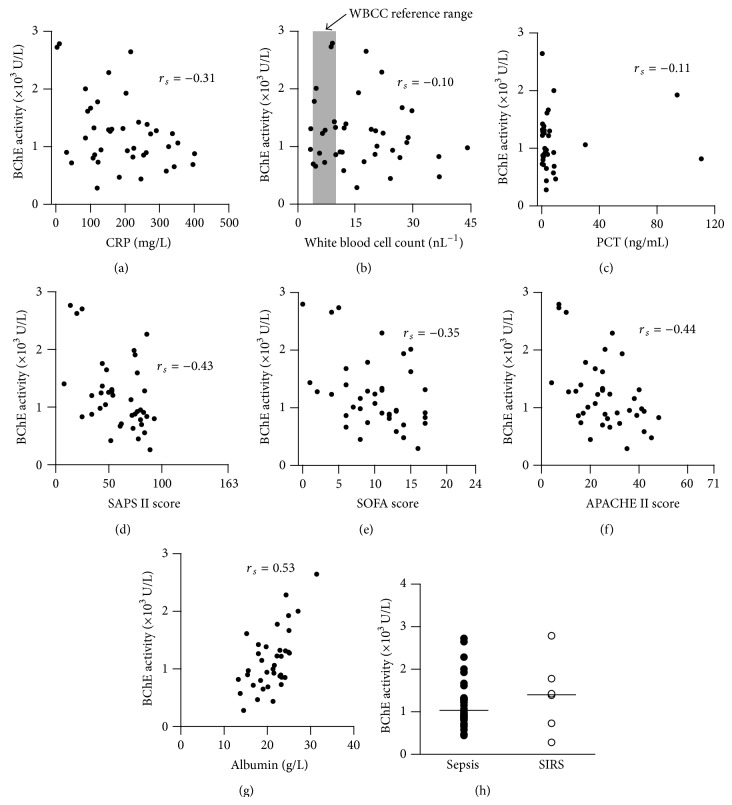
BChE activity correlates with the severity of systemic inflammation. Scatter plots illustrate a correlation between BChE activity and CRP level (*n* = 40 measurements (a)) and white blood cell count (WBCC, *n* = 40 measurements) (b) measured from patients with systemic inflammation. Shaded box in diagram (b) denotes laboratory reference interval (healthy limits) used for the WBCC. The analysis revealed no correlation between BChE activity and PCT concentration (*n* = 34 measurements (c)). BChE activity inversely correlates with SAPS II (d), SOFA (e), and APACHE II (f) disease severity scores (*n* = 40 measurements each). Panel (g) indicates strong direct correlation between serum albumin level, a disease severity biomarker, and BChE activity obtained from patients with severe systemic inflammation (*n* = 40 measurements). Histogram (h) represents BChE activity measurements from patients with diagnosed sepsis (closed circles) and those with SIRS (inflammation without proven infection, open circles). Bars are median values. *r*_*s*_: Spearman correlation coefficient; SAPS: simplified acute physiology score; SOFA: sequential organ failure assessment; APACHE: acute physiology and chronic health evaluation; SIRS: systemic inflammatory response syndrome.

We next asked whether BChE activity correlates with the degree of inflammation. The level of the BChE activity was compared to the level of the inflammatory biomarkers (CRP, WBCC, and PCT). The correlation analysis revealed negative correlation between the BChE activity and CRP concentration measured in patients with systemic inflammation (*r*_*s*_ = −0.31, Spearman's rank correlation test, Figure 2(a)). As expected, WBCC did not correlate with the BChE activity (*r*_*s*_ = −0.1, Spearman's rank correlation test, Figure 2(b)), presumably due to the bimodal distribution of the WBCC during the immune response (leukocytosis and leukocytopenia). PCT concentration did not correlate with the BChE activity (*r*_*s*_ = −0.11, Spearman's rank correlation test, Figure 2(c)).

Furthermore we examined the correlation between the BChE activity and the severity of the inflammation. A negative correlation was observed when SAPS II (*r*_*s*_ = −0.43), SOFA (*r*_*s*_ = −0.35), and APACHE II (*r*_*s*_ = −0.44) scores were compared to the BChE activity (Spearman's rank correlation test, Figures 2(d), 2(e), and 2(f)). Interaction between the BChE activity and the disease severity scores, both consecutively measured in patients during the course of systemic inflammation (*n* = 20 patients), is further illustrated in the supplementary Figure  1 in the Supplementary Material available online at http://dx.doi.org/10.1155/2015/274607.

Albumin concentration has been used as a disease severity marker. Reduced serum albumin concentration is associated with increased mortality in patients with critical illness [26, 27]. We compared the serum albumin concentration with the activity of BChE, obtained from ICU patients. Indeed, a positive correlation between serum albumin and BChE activity was observed, despite the fact that 8 patients (20%) with severe hypoalbuminemia (plasma albumin < 20 g/L) received albumin substitution (*r*_*s*_ = 0.53, Spearman's rank correlation test, Figure 2(g)).

In summary, these results reveal a correlation between BChE activity and both CRP and albumin concentrations observed during inflammation as well as strong correlation with disease severity scores (SAPS II, APACHE II, and SOFA). These results suggest that BChE activity might reflect systemic gradual changes during inflammation and serves as a marker of systemic inflammation severity.

**Figure 3:**
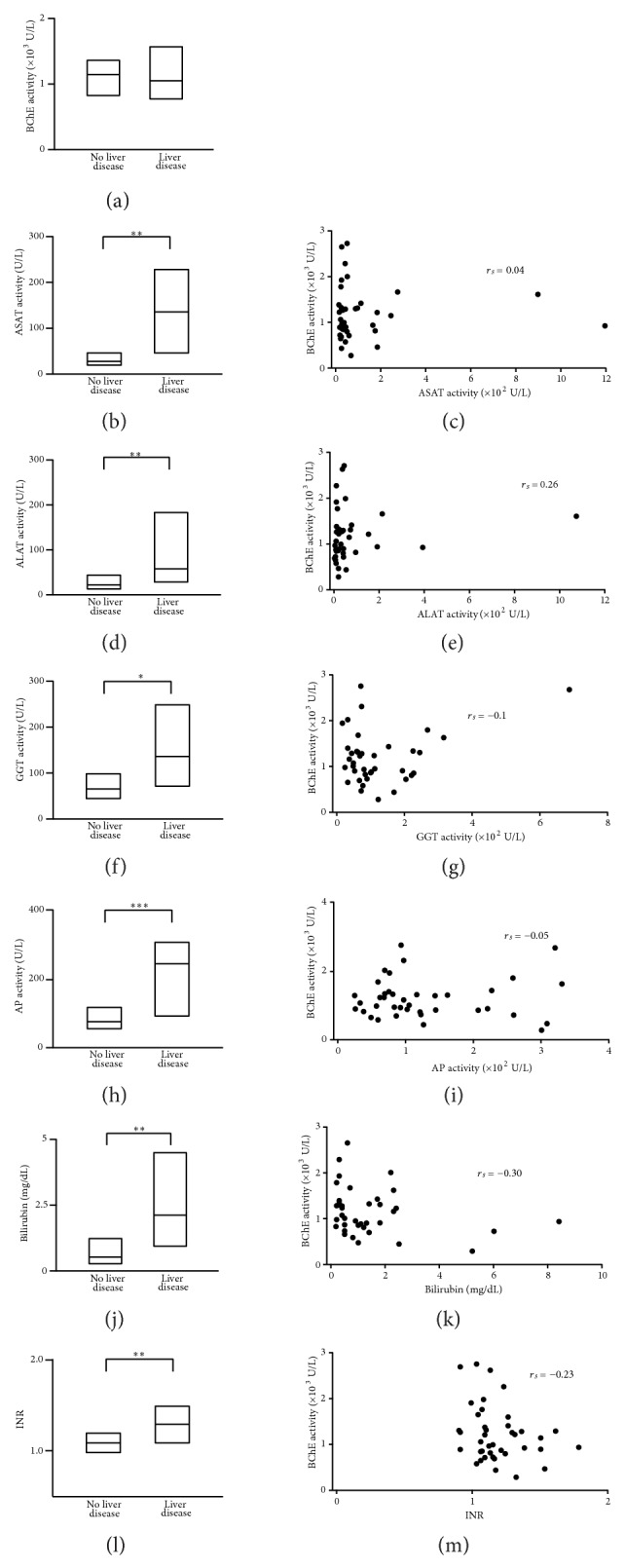
POCT-measured BChE activity during systemic inflammation remains disrupted, independent of the patient liver function. Box plots represent median values and 25%–75% percentile of the POCT-measured BChE activity (*n* = 40 measurements (a)) and standard laboratory tests for ASAT (*n* = 39 measurements (b)), ALAT (*n* = 39 measurements (d)), GGT (*n* = 39 measurements, f), AP (*n* = 39 measurements (h)) activity, total serum bilirubin concentration (*n* = 38 measurements (j)), and INR coagulation test (*n* = 40 measurements (l)) in patients with systemic inflammation associated with (liver disease) or without (no liver disease) hepatic dysfunction. Scatter plots represent the correlation degree between POCT-measured BChE activity and the corresponding ASAT (c), ALAT (e), GGT (g), AP (i) activity, bilirubin concentration (k), and INR (m). ASAT: aspartate aminotransferase; ALAT: alanine aminotransferase; GGT: gamma glutamyl transpeptidase; AP: alkaline phosphatase; INR: international normalized ratio. ^*^ *P* < 0.05, ^**^ *P* < 0.01, and ^***^ *P* < 0.001 (Mann-Whitney test); *r*_*s*_: Spearman correlation coefficient.

BChE is an enzyme produced in liver. Cholinesterase activity has been conventionally used as a liver function biomarker. Reduced cholinesterase activity is proposed to be the result of its disrupted synthesis in the liver. We investigated whether all patients with reduced BChE activity suffered from liver dysfunction. Based on standard laboratory liver function test results (ALAT, ASAT, GGT, AP, bilirubin, and INR) we divided patients into no-liver-disease and liver-disease subgroups. The criteria for “liver-disease group” included patients who have previously had liver surgery or patients with 3 or more of the following laboratory results: ALAT > 100 U/L, ASAT > 100 U/L, GGT > 100 U/L, AP > 200 U/L, total bilirubin > 2 mg/dL, and INR > 1.3. Observed reduction in the BChE activity was comparable in both subgroups (no-liver-disease: 1144 (544) U/L versus liver disease: 1048 (804) U/L, *P* = 0.79, Mann-Whitney test, Figure 3(a)) suggesting that BChE activity does not vary with liver disease. We further examined the correlation between BChE activity and each of the liver function parameters (Figures 3(b)–3(m)). As expected, the no-liver disease and liver disease groups differed for each of the liver function parameters (ASAT: 28 (26) U/L versus 135 (181) U/L, *P* < 0.01; ALAT: 24 (31) U/L versus 58 (156) U/L, *P* < 0.01; GGT: 69 (54) U/L versus 136 (178) U/L, *P* < 0.05; AP: 82 (62) U/L versus 243 (212) U/L, *P* < 0.001; bilirubin: 0.5 (0.9) mg/dL versus 2.1 (3.6) mg/dL, *P* < 0.01; INR: 1.1 (0.2) versus 1.3 (0.4), *P* < 0.01, Mann-Whitney test, Figures 3(b), 3(d), 3(f), 3(h), 3(j), and 3(l)). However, BChE activity did not correlate significantly with any of these liver function parameters (BChE versus ASAT: *r*_*s*_ = 0.04; BChE versus ALAT: *r*_*s*_ = 0.26; BChE versus GGT: *r*_*s*_ = −0.1; BChE versus AP: *r*_*s*_ = −0.05; BChE versus bilirubin: *r*_*s*_ = −0.3; BChE versus INR: *r*_*s*_ = −0.23, Spearman's rank correlation test, Figures 3(c), 3(e), 3(g), 3(i), 3(k), and 3(m)). Although hepatic dysfunction is often comorbid in patients with systemic inflammation, its occurrence and severity are not associated with sepsis-induced alterations in BChE activity.

Reduced BChE activity occurs irrespective of the type or origin of the systemic inflammation. Analysis of the BChE activity levels between patients with diagnosed sepsis and those with SIRS (inflammation without proven infection) revealed no differences (septic: 1032 (474) U/L versus SIRS: 1405 (1414) U/L, *P* = 0.45, Mann-Whitney test, Figure 2(h)). The definitive diagnosis of the sepsis, thus, remains a multiple diagnostic approach.

**Figure 4:**
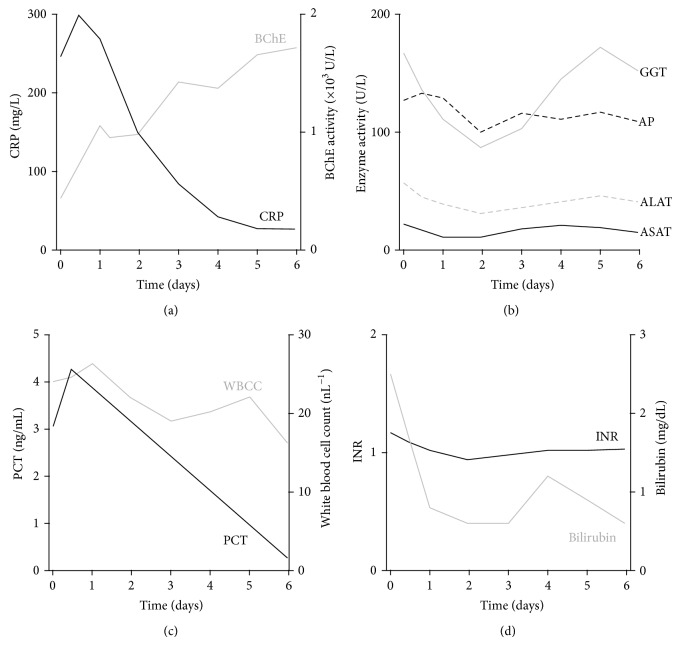
BChE activity mirrors the inflammatory biomarker dynamic change during the course of disease. Diagrams illustrate the course of the systemic inflammation in sixty-five-year-old patient with an abdominal nonhepatic septic focus over the time course of six days. Elevation in the BChE activity matches the drop of the CRP (a) and procalcitonin (PCT (c)) concentration and the fall in the white blood cell count (WBCC (c)). Note that hepatic function tests ASAT, ALAT, GGT, and AP in panel (b) and bilirubin and INR in (d) remained unaltered during the observed time period.

Interaction between BChE activity and inflammatory biomarkers but a lack of correlation with liver function can be further illustrated by a case of a patient admitted to the ICU with nonhepatic inflammatory disease. In a six-day course of disease with measurements of BChE, CRP (Figure 4(a)), PCT, and WBCC (Figure 4(c)) as well as liver function tests (Figures 4(b) and 4(d)) we observed a strong relationship between BChE activity and the inflammatory biomarkers, whereas liver function tests remained unaffected, further supporting the finding that the BChE activity might play an important role in the diagnosis of the systemic inflammation independent of overall hepatic function.

## 4. Discussion

We report that during systemic inflammation BChE shows dramatically reduced activity as compared to that in healthy individuals. Observed reduction in BChE activity is associated with elevated levels of inflammatory biomarkers. In addition, a negative correlation between BChE activity and the serum concentration of CRP has been detected. Furthermore, we observed a marked further reduction in the BChE activity in nonsurvivor group in comparison to the survivors. This finding is concordant with the highly elevated disease severity scores (e.g., APACHE II, SAPS II, and SOFA) obtained from nonsurvivor, as compared to the survivor group. This finding has been supported by a correlation analysis between the level of the serum BChE activity and the resulting disease severity scores, a well-established protocol used to evaluate and predict the severity of the disease in ICU patients [28–30]. BChE, therefore, might play an important role in indicating a systemic inflammation, as well as estimating the severity of the inflammatory disease and predicting its outcome/patient survival. In particular, gauging severity and predicting outcome are poorly indicated by conventional inflammatory biomarkers currently in use [31, 32].

This study demonstrates the potential value of BChE in a process of disease severity assessment and compares favorably with the work of Distelmaier and colleagues where BChE has been suggested to be a strong and independent inverse predictor of all-cause and cardiovascular mortality in patients undergoing venoarterial ECMO therapy following cardiovascular surgery [33].

Our findings complement those of Lampón and coworkers [34] where serum BChE activity correlated negatively with CRP concentration in the group of patients with acute inflammation. Patients included in the study were diagnosed with subclinical chronic low-grade systemic inflammation. Our results revealed a comparable negative correlation between BChE activity and systemic inflammation. However, CRP concentrations in our study were dramatically higher.

The nervous system interacts with the immune system in a bidirectional way. Acetylcholine (Ach) plays a crucial role in the neuroimmune interaction by acting anti-inflammatory [5]. BChE, an enzyme that hydrolyses acetylcholine, could control the cholinergic activity in a negative feedback loop manner. Reduced serum BChE might cause an increased Ach activity, which, in turn, results in enhanced anti-inflammatory systemic response. Some preclinical data lends weight to this hypothesis; physostigmine (blood-brain barrier permeable cholinesterase inhibitor) treatment improved survival after experimental endotoxemia [8]. This hypothesis would in turn predict that physostigmine administration during systemic inflammation might improve outcome in the early phase of sepsis therapy by enhancing cholinergic anti-inflammatory action. This however remains to be tested.

Measuring BChE activity may also provide an indirect estimate of the systemic response to a noxious stimulus allowing ICU staff to promptly respond with an appropriate therapy. Given that reduced BChE levels in patients with systemic inflammation did not differ between septic and SIRS patients and also were unaffected by liver comorbidity in our study, assessing BChE levels is unlikely to help identify the type of pathogen underlying the inflammation.

Indeed, our results showed that BChE activity in the inflammatory disease did not correlate with the level of procalcitonin. The cause of this discrepancy might be the fact that the etiopathology of the inflammatory disease in our patients has not been considered. No conclusion can be drawn whether the activity of BChE might identify septic versus sterile systemic inflammation. However, minimizing time delays between the clinical assessment and therapy of the systemic inflammation by means of the POCT approach could significantly improve the treatment of critically ill patients.

BChE is synthesized in the liver. Even though a physiological role of the BChE has not yet been identified, the activity of this enzyme strongly depends on liver function [35]. In contrast, we observed that activity levels of BChE in patients with no liver associated systemic inflammation varied irrespective of hepatic function. The reason for this may be that normal liver function is necessary for the BChE to be produced; however, the enzyme activity might be controlled by separate mechanisms. Acetylcholinesterase is a highly selective acetylcholine hydrolyzing enzyme, primarily active in central synapses and neuromuscular junctions, but only sparsely present outside the synapses [16, 36]. In contrast to acetylcholinesterase, BChE is an enzyme abundant in serum, which might be responsible for the nonsynaptic, presumably anti-inflammatory function of the acetylcholine in the negative-feedback loop manner.

Concerns have been raised regarding the validity of the BChE as an independent indicator for inflammation. The activity of BChE might, indeed, be affected during hepatic dysfunction; however, only a combined diagnostic approach, comprising various laboratory tests and clinical examinations would identify the most effective treatment decision.

Using an enzymatic assay to measure BChE raises the questions of possible interactions with serum protein contents or resuscitation fluids (e.g., hydroxyethyl starch (HES)). No relevant interference of this enzymatic assay with serum protein contents has been reported. Reversible cholinesterase antagonists (huperzine, donepezil, and galantamine) may cause reduced cholinesterase activity in serum. However, patients included in this study did not receive any cholinesterase inhibitors in the therapy. The management of septic patients in the intensive care unit includes early goal-directed therapy, as suggested in The International Guidelines for Management of Severe Sepsis and Septic Shock: Surviving Sepsis Campaign [19]. The guideline recommends against the use of hydroxyethyl starches (HES) for fluid resuscitation of severe sepsis and septic shock (grade 1B recommendation). Accordingly, our patients did not receive HES during fluid resuscitation. Therefore, we have no data concerning possible interference of the BChE assay with HES as resuscitation fluid.

The work of Assayag et al. has shown that postischemic stroke patients with lower serum BChE activity, or those with rare BChE mutations rendering lower hydrolytic activity show poorer recovery. This finding suggests that cholinesterase activity might predict the risk of poststroke mortality more effectively than inflammatory biomarkers [37]. None of the patients included in our study suffered an ischemic stroke; nevertheless, our results support a hypothesis of a tight correlation between cholinergic activity and the inflammatory response. Inversely, patients with metabolic syndrome have been shown to express increased BChE activities, such that reductions in their BChE might be masked [38]. The BChE activity was not measured prior to inflammation; therefore, we cannot exclude the possibility that patients with metabolic syndrome might show false elevated BChE activity levels during the systemic inflammatory disease. Cardiac patients under intense risk of reoccurring major adverse cardiac events (MACE) have been shown to express reduced BChE activity [39]. Patients with the acute cardiac event or with clinical suspicion thereof are biochemically tested for cardiac biomarkers in our ICU. None of the included patients suffered MACE prior to or during the study period.

This study has certain limitations that need to be taken into consideration. Patients lacked baseline BChE results prior to systemic inflammation and admission to the ICU. We cannot ascertain to what extent inflammation was responsible for the observed reduction of the BChE activity. On the basis of the present data it was not possible to distinguish the etiology of the systemic inflammatory disease without implementation of the microbiological analysis. Common mutations in the BChE gene could cause reduced BChE activity on inherited basis [40, 41] which may confound the hereby discussed findings. Impaired hepatic function imposes a constraint in the BChE activity interpretation regarding the inflammatory disease. Measuring changes in the activity of the BChE related to the loss of body fluids (ascites) and regarding blood transfusion would be of further interest. The kinetics of the enzyme activity regarding onset of the inflammation remains to be determined.

Finally, this study provides insight into the potential value of the POCT method to determine BChE activity. This is a simple test providing a rapid result in contrast with the significant time delay associated with the conventional laboratory analysis. The combination of goal-directed therapy and the POCT approach has been proven to dramatically decrease the mortality of patients undergoing congenital heart surgery [42]. Early recognition of inflammation is essential for the successful treatment of patients with suspected sepsis and thus potential delays in laboratory tests should be avoided to ensure prompt initiation of antibiotic treatment and early goal directed therapy.

## 5. Conclusions

We conclude that BChE activity is significantly reduced during systemic inflammation and that this reduction correlates with the disease severity. Although hepatic function has previously been found essential for BChE production, we show here that the reduced BChE activity associated with systemic inflammation occurs independently of and is thus not caused by any deficit in hepatic function in these patients. Therefore BChE activity might play an important role in the diagnosis of the systemic inflammation independent of overall hepatic function. POCT approach, used to assess BChE activity, might further improve the therapy of the critically ill patients by minimizing time delays inherent with lengthy laboratory testing of other conventional markers of sepsis. Measuring BChE activity might prove beneficial in recognition and early commencement of anti-inflammatory therapy, a decision particularly important in an ICU environment.

## Supplementary Material

Supplementary figure 1. Interaction between BChE activity and disease severity scores during the course of the systemic inflammation. Diagrams illustrate the dynamic changes of the BChE activity (red line) and the disease severity scores (APACHE II: black line; SOFA: black dotted line; SAPS II: black dashed line) over the time course of 2-6 days. Note that only patients who were consecutively monitored for both BChE activity and for the disease severity scores for more than 2 days were included (n = 20 patients). BChE: butyrylcholinesterase; SAPS: simplified acute physiology score; SOFA: sequential organ failure assessment; APACHE: acute physiology and chronic health evaluation.

## Figures and Tables

**Table 1:** Demographics and the clinical data of study population.

Demographic data	
Number of volunteers	40
Age^*^	38 (29–54)
Male sex	23 (58%)
Number of patients	40
Age^*^	67 (62–73)
Male sex	28 (70%)
Primary focus of systemic inflammation	
Gastrointestinal tract	21 (53%)
Lung	11 (27%)
Genitourinary tract	2 (5%)
SIRS	6 (15%)
Infection and inflammation parameters	
CRP (mg/L)^*^	189 (111–263)
PCT (ng/mL)^*^	2.7 (0.9–5.9)
WBC (nL^−1^)^*^	14 (7.4–24)
Outcome	
Survivor 28 days	31 (78%)
Disease severity scoring	
SAPS^*^	62 (44–80)
SOFA^*^	11 (6.3–14)
APACHE II^*^	25 (17–35)

SIRS: systemic inflammatory response syndrome, CRP: C-reactive protein, PCT: procalcitonin, WBCC: white blood cell count, SAPS: simplified acute physiology score, SOFA: the sequential organ failure assessment score, APACHE II: acute physiology and chronic health evaluation II, ^*^median with interquartile range.
