# Supplementary material for: Reduced Serum Butyrylcholinesterase Activity Indicates Severe Systemic Inflammation in Critically Ill Patients
Source: Mediators Inflamm. 2015 Feb 11;2015:274607. doi: 10.1155/2015/274607 (PMC4339712; doi:10.1155/2015/274607)

Supplementary figure 1

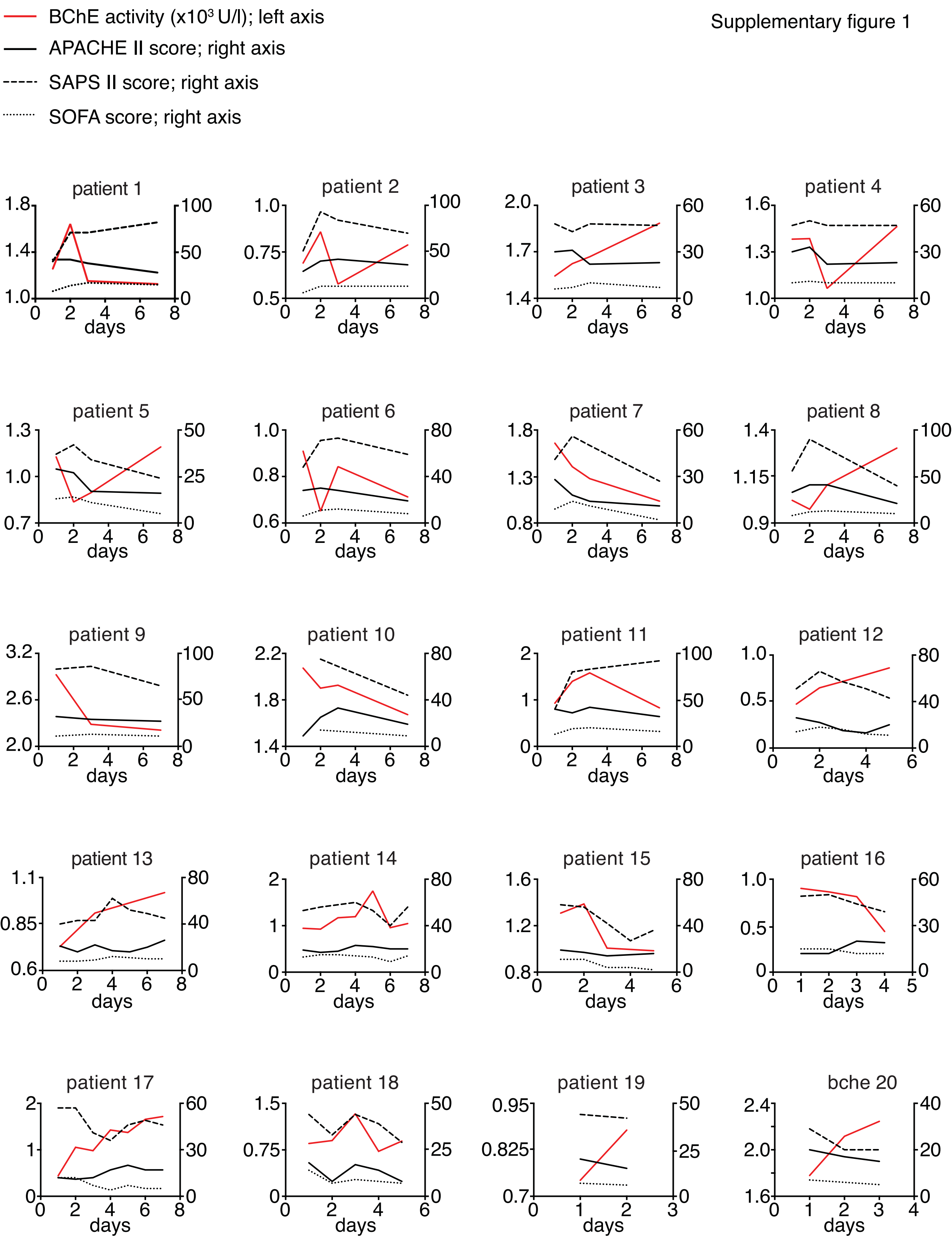

Supplement: Supplementary file 1 — Supplementary figure 1. Interaction between BChE activity and disease severity scores during the course of the systemic inflammation. Diagrams illustrate the dynamic changes of the BChE activity (red line) and the disease severity scores (APACHE II: black line; SOFA: black dotted line; SAPS II: black dashed line) over the time course of 2-6 days. Note that only patients who were consecutively monitored for both BChE activity and for the disease severity scores for more than 2 days were included (n = 20 patients). BChE: butyrylcholinesterase; SAPS: simplified acute physiology score; SOFA: sequential organ failure assessment; APACHE: acute physiology and chronic health evaluation. [file 274607.f1.pdf]
